# Lower Bounds on the Noiseless Worst-Case Complexity of Efficient Global Optimization

**DOI:** 10.1007/s10957-024-02399-1

**Published:** 2024-03-11

**Authors:** Wenjie Xu, Yuning Jiang, Emilio T. Maddalena, Colin N. Jones

**Affiliations:** 1 Automatic Control Laboratory, École Polytechnique Fédérale de Lausanne (EPFL), Lausanne, Switzerland; 2 Urban Energy Systems Laboratory, Swiss Federal Laboratories for Materials Science and Technology (Empa), Dübendorf, Switzerland

**Keywords:** Efficient global optimization, Worst-case complexity, Reproducing Kernel Hilbert space

## Abstract

Efficient global optimization is a widely used method for optimizing expensive black-box functions. In this paper, we study the worst-case oracle complexity of the efficient global optimization problem. In contrast to existing kernel-specific results, we derive a unified lower bound for the oracle complexity of efficient global optimization in terms of the metric entropy of a ball in its corresponding reproducing kernel Hilbert space. Moreover, we show that this lower bound nearly matches the upper bound attained by non-adaptive search algorithms, for the commonly used squared exponential kernel and the Matérn kernel with a large smoothness parameter $\nu$. This matching is up to a replacement of *d*/2 by *d* and a logarithmic term $\log \frac{R}{\epsilon }$, where *d* is the dimension of input space, *R* is the upper bound for the norm of the unknown black-box function, and $\epsilon$ is the desired accuracy. That is to say, our lower bound is nearly optimal for these kernels.

## Introduction

Black-box optimization by sequentially evaluating different candidate solutions without access to gradient information is a pervasive problem. For example, tuning the hyperparameters of machine learning models [3, 30], optimizing control system performance [2, 40] and discovering drugs or designing materials [10, 21], etc., can all be formulated as a black-box optimization problem without explicit gradient information. Therefore, efficient global optimization[13, 29], as a sample-efficient method to solve the expensive black-box optimization problem without explicit gradient information, has recently been receiving much attention. Efficient global optimization is based on the idea of constructing a surrogate function using Gaussian process regression or kernel ridge regression to guide the search of optimal solution [13].

In many applications, e.g., tuning the hyperparameters of a deep neural network (where the objective function in discrete variables, such as number of layers, can be regarded as a restriction of continuous function), each sample can take significant resources such as time and computation. For such problems, understanding the sample complexity of efficient global optimization is of great theoretical interest and practical relevance.

There is a large body of literature on the convergence rates of particular efficient global optimization algorithms [7, 26, 31, 33, 34, 37]. Two typical analysis set-ups are the Bayesian and non-Bayesian settings.^1^ In the Bayesian setting, the black-box function is assumed to be sampled from a Gaussian process, whereas in the non-Bayesian setting, the black-box function is assumed to be regular in the sense of having a bounded norm in the corresponding reproducing kernel Hilbert space.

As a complement to convergence analysis of different algorithms, complexity analysis tries to understand the inherent hardness of a problem. Specifically, we are interested in answering the question: *for a class of optimization problems, how many queries to an oracle, which returns some information about the function, are necessary to guarantee the identification of a solution with objective value at most *$\epsilon$ *worse than the optimal value* [22]? Without a complexity analysis, we cannot tell whether existing algorithms can be improved further in terms of convergence rate. This problem is well studied for convex optimization (e.g., in [22]), but less well understood for efficient global optimization.

Intuitively, the complexity of efficient global optimization largely depends on the richness or complexity of the functions inside the corresponding reproducing kernel Hilbert space (RKHS). Indeed, selecting the proper RKHS or the kernel function *k* is an important research question in the literature [14, 15]. Intuitively, the choice of the kernel functions captures the prior knowledge on the black-box function to optimize. As an extreme example, if we know the ground-truth black-box function is linear, we can adopt the linear kernel. Then after a finite number of noiseless function evaluations, we can uniquely determine the ground-truth function and hence the optimal solution. However, agnostically selecting simple kernels may lead to a surrogate function that is not expressive enough. For example, when the black-box function is nonlinear, using an RKHS with a linear kernel can not learn the ground-truth function well. For such a function, it is more reasonable to select a more expressive kernel such as the squared exponential kernel. To measure the complexity of a set of functions, *metric entropy* [16] is widely used in learning theory. However, as far as we know, the explicit connection between a complexity measure such as metric entropy for a function set and the problem complexity of efficient global optimization has not been established.

This paper focuses on the complexity analysis of efficient global optimization with general kernel functions in the non-Bayesian and noiseless setting. Although the noisy setting is more realistic from the practical point of view, it is also critical to consider the noiseless setting from the complexity-theoretic point of view. The rationale is that the noise may introduce additional statistical complexity to the problem and corrupt the inherent complexity analysis of the efficient global optimization. In addition, the noiseless setting is not a simple extension of the noisy setting. Existing analysis under noisy setting (e.g., [5, 25, 27, 28]) typically relies on strictly positive noise variance. Simply setting noise variance to zero makes the analysis and results diminish. For example, the noisy bound for Squared Exponential (SE) kernel in [28] is $\varOmega (\frac{\sigma ^2}{\epsilon ^2}\left( \log \frac{{R}}{\epsilon })^{\frac{d}{2}}\right)$, which is dominated by $\frac{\sigma ^2}{\epsilon ^2}$, where $\sigma ^2$ is the noise variance, $\epsilon$ is the desired accuracy,^2^ and *R* is the function norm upper bound. Simply setting $\sigma =0$ gives a meaningless $\varOmega (0)$ bound. Without the analysis under noiseless setting, it is unclear whether this dominant $\frac{\sigma ^2}{\epsilon ^2}$ term is due to noise or the inherent complexity of the RKHS.

**Table 1:** A summary of the state-of-the-art complexity result for efficient global optimization

Works	Noise	SE kernel	Matérn kernel	General kernel
[4]	No	N/A	$\varOmega \left( (\frac{1}{\epsilon })^{\frac{d}{\nu }}\right)$	N/A
[28]	Yes	$\varOmega \left( \frac{\sigma ^{2}}{\epsilon ^{2}}\left( \log \frac{R}{\epsilon }\right) ^{d / 2}\right)$	$\varOmega \left( \frac{\sigma ^{2}}{\epsilon ^{2}}\left( \frac{R}{\epsilon }\right) ^{d / \nu }\right)$	N/A
Ours	No	$\varOmega \left( \left( \log \frac{R}{\epsilon }\right) ^{d / 2-1}\right)$	$\varOmega \left( \frac{\left( \frac{R}{\epsilon }\right) ^{\frac{d}{\nu +d / 2}}}{\log \frac{R}{\epsilon }}\right)$	$\varOmega \left( \frac{\log \mathcal {N}\left( S(\mathcal {X}), 4 \epsilon ,\Vert \cdot \Vert _{\infty }\right) }{\log \left( \frac{R}{\epsilon }\right) }\right)$

$\sigma ^2$ is the noise variance. *R* is the function norm upper bound. *d* is the dimension of input space. $\nu$ is the smoothness parameter of Matérn kernel. N/A means ‘not applicable’. $S(\mathcal {X})$ is the ball in the corresponding reproducing kernel Hilbert space, with input set $\mathcal {X}$. $\mathcal {N}(\cdot , \cdot , \cdot )$ is the standard covering number to be formally defined in Sect. 4

To highlight our originality and contribution, a comparison of our results with the state-of-the-art complexity analysis is given in Table 1. As far as we know, our work is the first to give a unified general lower bound in terms of metric entropy. Interestingly, we also notice that the commonly seen $\varTheta (1/\epsilon ^2)$ term in the noisy setting disappears in the noiseless setting, which matches our intuition that estimating a point with Gaussian noise typically takes $\varTheta (1/\epsilon ^2)$ sample complexity. Specifically, our contributions include:

- We introduce a new set of analysis techniques and derive a *general* unified lower bound for the deterministic oracle complexity of efficient global optimization in terms of the *metric entropy* of the function space ball in the corresponding reproducing kernel Hilbert space, providing a unified and intuitive understanding for the complexity of efficient global optimization.
- Our *general* lower bound allows us to leverage existing estimates of the covering number of the function space ball in the RKHS to derive kernel-specific lower bounds for the commonly used squared exponential kernel and Matérn kernel with a large smoothness parameter $\nu$, without the commonly seen $1/\epsilon ^2$ term for the noisy setting interestingly. Furthermore, the lower bound for squared exponential kernel under noiseless setting is derived for the first time, to the best of our knowledge.
- We further show that these kernel-specific lower bounds nearly match the upper bounds attained by some non-adaptive search algorithms, where the upper bound for the squared exponential kernel is newly derived in this paper. Hence, our general lower bound is close to optimal for these specific kernels.

## Related Work

There has been a large body of literature on analyzing the complexity and the convergence properties of efficient global optimization. We first summarize the relevant literature area by area. We then highlight the position and the original contribution of our paper.

*Algorithm-dependent Convergence Analysis.* One line of research analyzes the properties of particular types of algorithms. For example, some papers [9, 17] analyze the consistency of efficient global optimization algorithms. Vazquez and Bect [34], Wang and de Freitas [37] analyze the convergence property of the expected improvement algorithm. Vakili et al. [33] proposes a maximum variance reduction algorithm that achieves optimal order simple regret for particular kernel functions. Under the assumption of Hölder continuity of the covariance function, lower and upper bounds are derived for the Bayesian setting in [12]. Among this set of literature, the works on information-theoretic upper bounds are more relevant to our metric entropy lower bound. Srinivas et al. [31] derives an information-theoretic upper bound for the cumulative regret of the upper confidence bound algorithm. Russo and Van Roy [26] gives an information-theoretic analysis of Thompson sampling. However, there is no existing work that provides a complementary information-theoretic lower bound.

*Kernel-specific Lower Bound Analysis.* As for lower bounds or complexity analysis, Bull [4] derives a lower bound of simple regret for Matérn kernel in a noise-free setting. Scarlett et al. [28] provides lower bounds of both simple regret and cumulative regret for the squared exponential and Matérn kernels. With the Matérn kernel, a tight regret bound has been provided for Bayesian optimization in one dimension in [27]. With heavy-tailed noise in the non-Bayesian setting, a cumulative regret lower bound has been provided for the Matérn and squared exponential kernels in [25]. More recently, Cai and Scarlett [5] provides lower bounds for both standard and robust Gaussian process bandit optimization. However, unlike the information-theoretic upper bound shown in [31], the existing lower bound results are mostly (if not all) restricted to specific kernel functions (mostly squared exponential and Matérn). The explicit connection between the optimization lower bound and the complexity of the RKHS has not been established so far in the existing literature. In this paper, we establish such a connection by constructing a lower bound in terms of *metric entropy*.

*Covering Number Estimate in RKHS.* Another area of research relevant to this paper is the estimate of covering number or metric entropy in function spaces. Some of the classical results are used in this paper. In [8, Sect. 3.3], the covering number for the function space ball in a Besov space is estimated. A technique to derive a lower estimate of the covering number for a stationary kernel is developed in [42], and as an application, a lower bound of a function space ball’s covering number for the squared exponential kernel is derived.

*General Information-based Complexity Analysis.* Our focus is efficient global optimization in this paper, due to its increasing popularity and lack of a unified and intuitive understanding for its complexity. Nevertheless, there have also been many classical works in the general area of information-based complexity analysis. For example, it is shown that the optimal convergence rates of global optimization are equivalent to those of approximation in the sup-norm [23]. However, approximation in the sup-norm itself is another hard problem with its complexity to be understood. There is also another set of results that try to connect the finite rank approximation, which is more general than sample-based interpolation, with metric entropy [8, 18, 32]. However, they can not be directly applied to our efficient global optimization problem, due to the general finite rank approximation definitions that are inconsistent with our sample-based efficient global optimization setting.

*Minimax Rates for Kernel Regression.* In learning theory, there are well-established results on covering number bound of learning errors. Many existing works [6, 24] derive covering number bounds for the generalization error of learning problems with RKHS or more general hypothetical spaces. However, in a typical learning setting, the sample points and corresponding observations are assumed to be identically and independently distributed, with observations corrupted by noise. To the contrast, the setting we consider in this paper is an essentially different global optimization problem. Specifically, our goal is to identify a solution with the desired level of optimality and the sample point can be adaptively selected.

*Position and Originality of Our Work.* Despite the rich literature summarized above, we notice two major limitations of the state-of-the-art complexity bounds. Firstly, existing analysis (see, e.g., [4, 5]) is typically restricted to a specific group of kernels (most commonly, the Squared Exponential kernel and the Matérn kernel). A unified understanding of the optimization complexity is lacking. Our work addresses this limitation by providing a unified general lower bound in terms of metric entropy, which recovers (close-to) state-of-the-art lower bounds when restricted to specific kernels. Secondly, the lower bounds with noise can be dominated by a $\varTheta \left( \frac{1}{\epsilon ^2}\right)$ term (e.g., in [28] for squared exponential kernel), which may corrupt the understanding for the complexity of efficient global optimization. Our work addresses this limitation by proving bounds in the noiseless regime.

## Problem Statement

We consider efficient global optimization in a non-Bayesian setting [31]. Specifically, we optimize a deterministic function *f* from a reproducing kernel Hilbert space (RKHS) $\mathcal {H}$ with input space $\mathbb {R}^d$, where *d* is the dimension. $\mathcal {H}$ is equipped with the reproducing kernel $k(\cdot , \cdot ):\mathbb {R}^d\times \mathbb {R}^d{\rightarrow }\mathbb {R}$. Let $\mathcal {X}\subset \mathbb {R}^d$ be the known feasible set (e.g., a hyperbox) of the optimization problem. In the following, we will use [*n*] to denote the set $\{1, 2, \ldots , n\}$. We assume that

### Assumption 3.1

$\mathcal {X}$ is compact and nonempty.

Assumption 3.1 is reasonable because, in many applications (e.g., continuous hyperparameter tuning) of efficient global optimization, we are able to restrict the optimization into certain ranges based on domain knowledge. Regarding the black-box function $f\in \mathcal {H}$ that we aim to optimize, we assume that,

### Assumption 3.2

$\Vert {f}\Vert _\mathcal {H}\le R$, where *R* is a positive real number and $\Vert \cdot \Vert _\mathcal {H}$ is the norm induced by the inner product associated with $\mathcal {H}$.

Assumption 3.2 requires that the function to be optimized is regular in the sense that it has bounded norm in the RKHS, which is a common assumption (e.g., [4, 28]) for complexity and convergence analysis.

### Assumption 3.3

$k(x_1,x_2)\le 1, \forall x_1,x_2\in \mathcal {X}$ and $k(x_1, x_2)$ is continuous on $\mathbb {R}^d\times \mathbb {R}^d$.

Assumption 3.3 is a common assumption for analyzing the convergence and complexity of efficient global optimization. It holds for a large class of commonly used kernel functions (e.g., Matérn kernel and squared exponential kernel) after normalization.

Our problem^3^ is formulated as

1 $$\begin{aligned} \min _{x\in \mathcal {X}}\quad f(x). \end{aligned}$$

We know that

$$\begin{aligned} f(x_1)-f(x_2)=\langle f, k(x_1,\cdot )-k(x_2, \cdot )\rangle \le \left\Vert f\right\Vert _\mathcal {H}\left\Vert k(x_1,\cdot )-k(x_2,\cdot )\right\Vert _\mathcal {H}. \end{aligned}$$

Hence, it can be shown under Assumptions 3.2 and 3.3, that *f* is continuous and thus (1) has an optimal solution on the compact set $\mathcal {X}$. As in standard efficient global optimization, we restrict ourselves to the zero-order oracle case. That is, our algorithm can only query the function value *f*(*x*) but not higher-order information at a point *x* in each step. Based on the function evaluations before the current step, the algorithm sequentially decides the next point to sample. In this paper, we only consider oracle query (namely, function evaluation) complexity without considering the complexity of solving auxiliary optimization problems in typical efficient global optimization algorithms (e.g., maximizing the expected improvement).

In this paper, we focus on the performance metric of *simple regret* $r_{(t)}$.

### Definition 3.1

*(Simple regret)* After *t* function evaluations, simple regret $r_{(t)}:=\min _{\tau \in [t]}f(x_\tau )-\min _{x\in \mathcal {X}}f(x)$, where $[t]:=\{1, 2,\ldots ,t\}$.

Note that in some of the literature, simple regret is also defined as $f({\hat{x}}_t)-\min _{x\in \mathcal {X}}f(x)$, where ${\hat{x}}_t$ is one additional point reported after *t* steps. Since we can always pay one more function evaluation for the reported point, this definition difference will not impact our convergence or complexity analysis.

## Preliminary

To analyze the problem complexity of efficient global optimization, we need a metric to measure the complexity of the RKHS. As an extreme example, if we choose a linear kernel, the underlying function to be optimized is a linear function. Hence, we can reconstruct it after a finite number of steps and compute the optimum without any error. The covering number is such a widely used metric to measure the complexity of an RKHS [41]. To facilitate our discussion, we introduce some concepts about the complexity of function sets.

Given a normed vector space $(V,\left\Vert \cdot \right\Vert )$ and a subset $G\subset V$, for $\epsilon >0$, we make the following complexity related definitions [39].

### Definition 4.1

($\epsilon$*-covering*) $\{v_1,\ldots ,v_N\}$ is an $\epsilon$-covering of $G$ if

$$\begin{aligned} G\subset \cup _{i\in [N]}B_{\left\Vert \cdot \right\Vert }(v_i,\epsilon ), \end{aligned}$$

where $B_{\left\Vert \cdot \right\Vert }(v_i,\epsilon )$ is the ball in *V* centered at $v_i$ with radius $\epsilon$ with respect to the norm $\left\Vert \cdot \right\Vert$.

### Definition 4.2

($\epsilon$*-packing*) $\{v_1,\ldots ,v_N\}\subset G$ is an $\epsilon$-packing of $G$ if

$$\begin{aligned} \min _{i\ne j}\left\Vert v_i-v_j\right\Vert >\epsilon . \end{aligned}$$

### Definition 4.3

*(Covering number)* The covering number $\mathcal {N}(G, \epsilon , \left\Vert \cdot \right\Vert )$ is defined to be $\min \left\{ n\,|\,\exists \epsilon \text {-covering } \{v_1,\ldots ,v_n\} \text { with cardinality } n\right\}$.

### Definition 4.4

*(Packing number)* The packing number $\mathcal {M}(G, \epsilon , \left\Vert \cdot \right\Vert )$ is defined to be $\max \left\{ n\,|\,\exists \epsilon \text {-packing } \{v_1,\ldots ,v_n\} \text { with cardinality } n\right\}$.

### Definition 4.5

*(Metric entropy)* The metric entropy of $(G, \left\Vert \cdot \right\Vert )$ is defined to be $\log \mathcal {N}(G, \epsilon , \left\Vert \cdot \right\Vert )$, where $\mathcal {N}$ is the covering number.

It can be verified that,

### Proposition 4.1

(Thm. IV, [16]) $$\mathcal {N}(G,\epsilon , \left\Vert \cdot \right\Vert )\le \mathcal {M}(G,\epsilon , \left\Vert \cdot \right\Vert )\le \mathcal {N}(G,\frac{\epsilon }{2}, \left\Vert \cdot \right\Vert )$$.

To facilitate the subsequent complexity analysis, we use $x_1, x_2,\ldots ,x_t$ to denote the sequence of evaluated points up to step *t*. We now formalize the concept of a deterministic algorithm for solving the efficient global optimization problem.

### Definition 4.6

*(Deterministic algorithm)* A deterministic algorithm $\mathcal {A}$ for solving the optimization problem in (1) is a sequence of mappings $(\pi _t)_{t=1}^\infty$, where $\pi _t:(\mathcal {X}\times \mathbb {R})^{t-1}{\rightarrow }\mathcal {X},t\ge 2$ and $\pi _1: \{\emptyset \}{\rightarrow }\mathcal {X}$. When running the algorithm $\mathcal {A}$, the sample at step *t* is $x_t=\pi _t((x_{\tau }, f(x_\tau ))_{\tau =1}^{t-1}),t\ge 2$ and $x_1=\pi _1(\emptyset )$.

Note that deterministic algorithms include most of the popular acquisition functions based efficient global optimization algorithms (e.g., lower/upper confidence bound [31] and expected improvement [13]).

We assume that the first sample point $x_1$ is deterministic, either given before running the algorithm or chosen by the algorithm. Now, if we suppose that *f* is such that the algorithm observes a sequence of 0’s for every function evaluation $f(x_\tau )$, it will generate a deterministic sample trajectory. We will see in our main result that this trajectory can be used to construct adversarial functions to derive the lower bound. We formally define it below.

### Definition 4.7

*(Zero sequence)* Given a deterministic algorithm $\mathcal {A}=(\pi _t)^\infty _{t=1}$. We set $x^0_1=\pi _1(\emptyset )$. Applying the recurrence relationship $x^0_t=\pi _t((x_\tau ^0, 0)_{\tau =1}^{t-1})$, we get a deterministic sequence $x_1^0, x_2^0,\ldots , x_t^0,\ldots$, which only depends on the algorithm $\mathcal {A}$. We call this sequence the zero sequence of the algorithm $\mathcal {A}$.

**Fig. 1:**
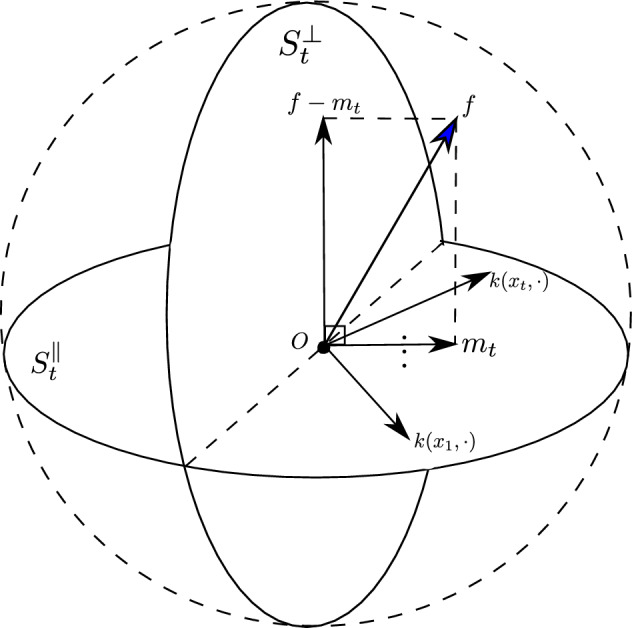
The function space view of our proof strategy

## Main Results

Our strategy to derive the lower bound is decomposing the RKHS into two orthogonal subspaces with one of them expanding as more samples are obtained, as shown in Fig. 1. Then, we can project the function space ball into these two subspaces. We will show that as the number of sampled points grows, the covering number of the ball’s projection into one subspace increases and the other decreases. We derive the lower bound on the number of optimization steps by bounding the increase/decrease rate. All the proofs of the lemmas and theorems are attached in the Appendix, except Lemma 5.4 and Theorem 5.1. Before proceeding, we introduce some notations.

**Notations** For $f\in \mathcal {H}$, $f|_\mathcal {X}:\mathcal {X}{\rightarrow }\mathbb {R}$ is defined as $f|_\mathcal {X}(x)=f(x),\forall x\in \mathcal {X}$. For $Q\subset \mathcal {H}$, we use $Q(\mathcal {X})$ to denote the set $\{f|_{\mathcal {X}}|f\in Q\}$, which is a subset of $C(\mathcal {X}, \left\Vert \cdot \right\Vert _\infty )$, the continuous function space over $\mathcal {X}$. $Q(\mathcal {X})$ is considered as a subset of $C(\mathcal {X}, \left\Vert \cdot \right\Vert _\infty )$ in $\mathcal {N}(Q(\mathcal {X}), \epsilon , \left\Vert \cdot \right\Vert _\infty )$ and $\mathcal {M}(Q(\mathcal {X}), \epsilon , \left\Vert \cdot \right\Vert _\infty )$.

We first decompose the RKHS into two orthogonal subspaces.

### Definition 5.1

$$\mathcal {H}_t^{\Vert }:=\{\sum _{i\in [t]}\alpha _ik(x_i,\cdot )|\alpha _i\in \mathbb {R}\},$$ $$\mathcal {H}_t^{\perp }:=\{f\in \mathcal {H}|f(x_i)=0,\forall i\in [t]\}$$.

Notice that $\mathcal {H}_t^\Vert$ expands when we have more and more function evaluation data. In parallel, $\mathcal {H}_t^\perp$ shrinks. We then consider the intersection of the function space ball *S* with $\mathcal {H}_t^\Vert$ and $\mathcal {H}_t^\perp$.

### Definition 5.2

$$S:=\{f|f\in \mathcal {H}, \left\Vert f\right\Vert _\mathcal {H}\le R\}, S_t^\Vert :=\mathcal {H}_t^\Vert \cap S, S_t^\perp :=\mathcal {H}_t^\perp \cap S$$.

With these definitions, we can show that any function in *S* can be decomposed into two functions in $S_t^\Vert$ and $S_t^\perp$, respectively.

### Lemma 5.1

$\forall f\in S$, there exists $m_t\in S_t^\Vert$, such that $f-{m}_t\in S_t^\perp$.

### Remark 5.1

When the matrix $K=(k(x_i,x_j))_{i,j\in [t]}$ is invertible, we can check that ${m}_t(x)=f_X^TK^{-1}K_{Xx}$, where $f_X=[f(x_1), f(x_2),\ldots , f(x_t)]^T$ and $K_{Xx}=[k(x_1,x), k(x_2,x), \cdots , k(x_t, x)]^T$, satisfies $m_t\in S_t^\Vert$ and $f-{m}_t\in S_t^\perp$. The function $m_t(x)$ is exactly the posterior mean function in Gaussian process regression.

Intuitively, we can add some function from $S_t^\perp$ to *f* without changing the historical evaluations at $x_1,\ldots ,x_t$. If we have some way of lower bounding the complexity of $S_t^\perp$, we may be able to find a perturbing function from $S_t^\perp$ that leads to suboptimality. We will try to lower bound the complexity of $S_t^\perp$ through Lemmas 5.2 and  5.3.

Since $S_t^\Vert$ and $S_t^\perp$ are orthogonal to each other in the RKHS, it is intuitive that the complexity of *S* can be decomposed into the complexity of $S_t^\perp$ and $S_t^\Vert$. Formally, we have Lemma 5.2.

### Lemma 5.2

For any $\epsilon _t^\Vert>0, \epsilon _t^\perp >0$, we have

$$\begin{aligned} \mathcal {M}(S_t^\perp (\mathcal {X}), \epsilon _t^\perp , \left\Vert \cdot \right\Vert _\infty )\ge \frac{\mathcal {N}(S(\mathcal {X}), \epsilon _t,\left\Vert \cdot \right\Vert _\infty )}{\mathcal {N}(S_t^\Vert (\mathcal {X}), \epsilon _t^\Vert ,\left\Vert \cdot \right\Vert _\infty )}, \end{aligned}$$

where $$\epsilon _t=\epsilon _t^\Vert +\epsilon _t^\perp $$.

Lemma 5.2 is proved based on Lemma 5.1. With Lemma 5.2, we can lower bound $\mathcal {M}(S_t^\perp (\mathcal {X}), \epsilon _t^\perp , \left\Vert \cdot \right\Vert _\infty )$ if we are able to upper bound $\mathcal {N}(S_t^\Vert (\mathcal {X}), \epsilon _t^\Vert ,\left\Vert \cdot \right\Vert _\infty )$.

Since $S_t^\Vert$ is inside a finite dimensional space $\mathcal {H}_t^\Vert$, we can show that,

### Lemma 5.3

If $0<\epsilon <\frac{R}{4}$, we have $\log \mathcal {N}{(S_t^\Vert (\mathcal {X}), \epsilon ,\left\Vert \cdot \right\Vert _\infty )}\le 2t\log \left( \frac{R}{\epsilon }\right)$.

We then give the following key lemma.

### Lemma 5.4

For $0<\epsilon <\epsilon _0$, if $t\le \frac{\log \mathcal {N}(S(\mathcal {X}), 4\epsilon ,\left\Vert \cdot \right\Vert _\infty )}{4\log \left( \frac{R}{\epsilon }\right) }$, then for any sample sequence $x_1, \cdots , x_t$, we have,

$$\begin{aligned} \frac{\mathcal {N}(S(\mathcal {X}), 4\epsilon ,\left\Vert \cdot \right\Vert _\infty )}{\mathcal {N}(S_t^\Vert (\mathcal {X}), \epsilon ,\left\Vert \cdot \right\Vert _\infty )}\ge 2, \end{aligned}$$

where $$\epsilon _0=\sup \left\{ \delta |\delta>0, \log \mathcal {N}(S(\mathcal {X}),4\delta , \left\Vert \cdot \right\Vert _{\infty })>2\log 2 \right\} $$.

### Proof

By assumption that $t\le \frac{\log \mathcal {N}(S(\mathcal {X}), 4\epsilon ,\left\Vert \cdot \right\Vert _\infty )}{4\log \left( \frac{R}{\epsilon }\right) }$, we have

$$\begin{aligned} 2t\log \left( \frac{R}{\epsilon }\right) \le \frac{1}{2}{\log \mathcal {N}(S(\mathcal {X}), 4\epsilon ,\left\Vert \cdot \right\Vert _\infty )}. \end{aligned}$$

By $\epsilon <\epsilon _0$ and the definition of $\epsilon _0$, $\frac{1}{2}{\log \mathcal {N}(S(\mathcal {X}), 4\epsilon ,\left\Vert \cdot \right\Vert _\infty )}-\log 2>0$. We also notice that $\log \mathcal {N}(S(\mathcal {X}),R, \left\Vert \cdot \right\Vert _{\infty })=0<2\log 2$ and thus, $\epsilon _0\le \frac{R}{4}$. We then can apply Lemma 5.3 to derive,

$$\begin{aligned}&\log \mathcal {N}({S}_t^\Vert (\mathcal {X}), \epsilon ,\left\Vert \cdot \right\Vert _{\infty })\le ~2t\log \left( \frac{R}{\epsilon }\right) \\&\quad \le ~\frac{1}{2}{\log \mathcal {N}(S(\mathcal {X}), 4\epsilon ,\left\Vert \cdot \right\Vert _\infty )}+\underbrace{\frac{1}{2}{\log \mathcal {N}(S(\mathcal {X}), 4\epsilon ,\left\Vert \cdot \right\Vert _\infty )}-\log 2}_{\text{ positive }}\\&\quad =~\log \mathcal {N}(S(\mathcal {X}), 4\epsilon ,\left\Vert \cdot \right\Vert _\infty )-\log 2, \end{aligned}$$

where the first inequality follows by Lemma 5.3 and the second by assumption on *t*. So $\frac{\mathcal {N}(S(\mathcal {X}), 4\epsilon ,\left\Vert \cdot \right\Vert _\infty )}{\mathcal {N}(S_t^\Vert (\mathcal {X}), \epsilon ,\left\Vert \cdot \right\Vert _\infty )}\ge 2$. $\square$

We are now ready to give our main result in Theorem 5.1.

### Theorem 5.1

If there exists a deterministic algorithm that achieves simple regret $r_{(T)}\le \epsilon$ for any function $f\in S$ in *T* function evaluations for our problem (1), it is necessary that,

2 $$\begin{aligned} T=\varOmega \left( \frac{\log \mathcal {N}(S(\mathcal {X}), 4\epsilon ,\left\Vert \cdot \right\Vert _\infty )}{\log (\frac{R}{\epsilon })}\right) . \end{aligned}$$

Before we prove Theorem 5.1, we give a sketch of the proof. For any deterministic algorithm and any number of optimization steps *t*, we consider the corresponding deterministic zero sequence $x^0_1, x^0_2, \cdots , x^0_t$ as defined in Definition 4.7. We try to construct an adversarial function inside the corresponding $S_t^\perp$ with 0 function value at the points $x^0_i,i\in [t]$ and low function values at some point that is not sampled. The possible minimal value of such an adversarial function links to the covering number of the set $S_t^\perp (\mathcal {X})$, which can be lower bounded by combining Lemmas 5.2 and  5.3.

### Proof of Theorem 5.1

Given an deterministic algorithm $\mathcal {A}=(\pi _t)_{t=1}^{+\infty }$, if it always gets the evaluations 0, then the sample trajectory satisfies,

$$\begin{aligned} x_t^0 = \pi _t\left( (x_\tau ^0,0)_{\tau =1}^{t-1}\right) , t\ge 2, \end{aligned}$$

which is exactly the zero sequence of the algorithm. Note that the zero sequence $x_t^0$ only depends on the deterministic algorithm $\mathcal {A}$. Once we fix the algorithm, the zero sequence is fixed.

We want to check the feasibility of the problem (3),

3 $$\begin{aligned} \underset{s \in \mathcal {S}, x\in \mathcal {X}}{ \min }\;\;~1\quad \text{ s.t. } \;\; \left\{ \begin{aligned}&s\left( x_{n}^0\right) =0,~\forall n=1, \ldots , t,\\&s(x)<-\epsilon . \end{aligned}\right. \end{aligned}$$

Any feasible solution of (3) has some ‘adversarial’ property against the algorithm $\mathcal {A}$. In fact, suppose that $({\bar{s}},{\bar{x}})$ is a feasible solution for problem (3), when we run the algorithm $\mathcal {A}$ over ${\bar{s}}$, the sample sequence up to step *t* is exactly the zero sequence truncated at step *t* and $r_{(t)}=\min _{\tau \in [t]}{\bar{s}}(x_\tau ^0)-\min _{x\in \mathcal {X}}{\bar{s}}(x)>\epsilon$. Now the question is under what condition, the problem (3) is feasible. Since we are analyzing the asymptotic rate, we restrict to the case $\epsilon <\epsilon _0$, where $\epsilon _0$ is given in Lemma 5.4. By Lemmas 5.4 and 5.2, if $t\le \frac{\log \mathcal {N}(S(\mathcal {X}), 4\epsilon ,\left\Vert \cdot \right\Vert _\infty )}{4\log \left( \frac{R}{\epsilon }\right) }$, for the sample sequence $x_1^0, \cdots , x_t^0$ corresponding to any given algorithm, we have,

$$\begin{aligned} \mathcal {M}(S_t^\perp (\mathcal {X}), 3\epsilon , \left\Vert \cdot \right\Vert _\infty )\ge \frac{\mathcal {N}(S(\mathcal {X}), 4\epsilon ,\left\Vert \cdot \right\Vert _\infty )}{\mathcal {N}(S_t^\Vert (\mathcal {X}), \epsilon ,\left\Vert \cdot \right\Vert _\infty )}\ge 2. \end{aligned}$$

Therefore, there exists functions $f_1, f_2\in S_t^\perp$, such that, $\left\Vert f_1|_\mathcal {X}-f_2|_\mathcal {X}\right\Vert _\infty \ge 3\epsilon$. So $\left\Vert f_1|_\mathcal {X}\right\Vert _\infty +\left\Vert f_2|_\mathcal {X}\right\Vert _\infty \ge \left\Vert f_1|_\mathcal {X}-f_2|_\mathcal {X}\right\Vert _\infty \ge 3\epsilon$ and at least one of $f_1$ and $f_2$ has $L_\infty$ norm over the set $\mathcal {X}$ at least $\frac{3\epsilon }{2}$. Without loss of generality, we assume $\left\Vert f_1|_\mathcal {X}\right\Vert _\infty \ge \frac{3\epsilon }{2}$. Since for $\forall g\in S_t^\perp$, $-g\in S_t^\perp$, there exists ${\hat{f}}\in S_t^\perp$ (either $f_1$ or $-f_1$), such that,

$$\begin{aligned} \inf _{x\in \mathcal {X}}{\hat{f}}(x)\le -\frac{3\epsilon }{2}. \end{aligned}$$

When applying the given algorithm to ${\hat{f}}$, if $t\le \frac{\log \mathcal {N}(S(\mathcal {X}), 4\epsilon ,\left\Vert \cdot \right\Vert _\infty )}{4\log \left( \frac{R}{\epsilon }\right) }$, the suboptimality gap or the simple regret $r_{(t)}$ is at least $\frac{3}{2}\epsilon$. Therefore, to reduce the simple regret $r_{(T)}\le \epsilon$ for all the functions in *S* within *T* steps, it is necessary that,

$$\begin{aligned} T=\varOmega \left( \frac{\log \mathcal {N}(S(\mathcal {X}), 4\epsilon ,\left\Vert \cdot \right\Vert _\infty )}{\log (\frac{R}{\epsilon })}\right) . \end{aligned}$$

$$\square $$

To verify the effectiveness of Theorem 5.1, we apply it to a simple case in Ex. 5.1.

### Example 5.1

For the quadratic kernel $k(x,y)=(x^Ty)^2$, the corresponding RKHS is finite-dimensional and is given as [20],

4 $$\begin{aligned} \mathcal {H}=\left\{ f_A(x)=x^TAx|A\in \mathcal {S}^{d\times d}\right\} , \end{aligned}$$

where $\mathcal {S}^{d\times d}$ is the set of symmetric matrices of size $d\times d$. We know that,

5 $$\begin{aligned} \langle f_{A_1}, f_{A_2}\rangle _\mathcal {H}= \langle A_1, A_2\rangle _\textrm{F}, \end{aligned}$$

where $\langle \cdot ,\cdot \rangle _\textrm{F}$ is the Frobenius inner product. Since $\mathcal {S}^{d\times d}$ can be embedded into $\mathbb {R}^{\frac{d\times (d+1)}{2}}$ and the metric entropy for compact set in Euclidean space is ${\varTheta }\left( \log \frac{1}{\epsilon }\right)$ as discussed in [39], the lower bound in Theorem 5.1 reduces to a constant. By applying a grid search algorithm for the quadratic kernel, we can identify the ground-truth function after a finite number of steps and determine the optimal solution without any error. Therefore, the lower bound is tight in $\epsilon$ for the quadratic kernel.

### Comparison with Upper Bounds for Commonly Used Kernels

Ex. 5.1 demonstrates the validity of Theorem 5.1 for simple quadratic kernel functions. In this section, we will derive kernel-specific lower bounds for the squared exponential kernel and the Matérn kernels by using Theorem 5.1 and existing estimates of the covering numbers for their RKHS’s. We compare our lower bounds with derived/existing upper bounds and show that they nearly match.

#### Squared Exponential Kernel

One widely used kernel in efficient global optimization is the squared exponential (SE) kernel given by

6 $$\begin{aligned} k(x,y) = \exp {\left\{ -\frac{\left\Vert x-y\right\Vert ^2}{\sigma ^2}\right\} }. \end{aligned}$$

In this case, we restrict to $\mathcal {X}=[0,1]^d$. By applying Theorem 5.1, we have,

##### Theorem 5.2

With $\mathcal {X}=[0,1]^d$ and using the squared exponential kernel, if there exists a deterministic algorithm that achieves simple regret $r_{(T)}\le \epsilon$ for any function $f\in S$ in *T* function evaluations for our problem (1), it is necessary that,

7 $$\begin{aligned} T=\varOmega \left( \left( \log \frac{R}{\epsilon }\right) ^{d/2-1}\right) . \end{aligned}$$

Furthermore, there exists a deterministic algorithm and *T* satisfying

$$\begin{aligned} T=\mathcal {O}\left( \left( \log \frac{R}{\epsilon }\right) ^{d}\right) \end{aligned}$$

such that the algorithm achieves $r_{(T)}\le \epsilon$ in *T* function evaluations for any $f\in S$.

The upper bound part is obtained through sampling non-adaptively to reduce the posterior variance to a uniform low level in $\mathcal {X}$. In this theorem, we focus on the asymptotic analysis of efficient global optimization and hide the coefficients that may depend on the dimension. We notice that the upper bound and lower bound are both polynomial in $\log \frac{1}{\epsilon }$ and nearly match, up to a replacement of *d*/2 by *d* in the order and one additional logarithmic term $\log \frac{R}{\epsilon }$.

#### Matérn Kernel

In this section, we consider the Matérn kernel,

8 $$\begin{aligned} k(x,y)=C_{\nu }(\left\Vert x-y\right\Vert )=\sigma ^{2} \frac{2^{1-\nu }}{\varGamma (\nu )}\left( \sqrt{2 \nu } \frac{\left\Vert x-y\right\Vert }{\rho }\right) ^{\nu } K_{\nu }\left( \sqrt{2 \nu } \frac{\left\Vert x-y\right\Vert }{\rho }\right) , \end{aligned}$$

where $\rho$ and $\nu$ are positive parameters of the kernel function, $\varGamma$ is the gamma function, and $K_{\nu }$ is the modified Bessel function of the second kind.

##### Theorem 5.3

With $\mathcal {X}=[0, 1]^d$ and the Matérn kernel, if there exists a deterministic algorithm that achieves simple regret $r_{(T)}\le \epsilon$ for any function $f\in S$ in *T* function evaluations for our problem (1), it is necessary that,

9 $$\begin{aligned} T=\varOmega \left( \left( \frac{R}{\epsilon }\right) ^{\frac{d}{\nu +d/2}}\left( \log \frac{R}{\epsilon }\right) ^{-1}\right) . \end{aligned}$$

Furthermore, there exists a deterministic algorithm and *T* satisfying,

10 $$\begin{aligned} T=\mathcal {O}\left( \left( \frac{R}{\epsilon }\right) ^{\frac{d}{\nu }}\right) , \end{aligned}$$

such that the algorithm achieves $r_{(T)}\le \epsilon$ in *T* function evaluations for any $f\in S$.

##### Remark 5.2

The upper bound part of Theorem 5.3 is proved by Theorem 1 of [4]. We also notice that [4] provides a lower bound of the same order as the upper bound in Eq. (10), which means that the upper bound order is also the optimal lower bound order.

##### Remark 5.3

When ${\nu }\ge \frac{1}{2}d$, our lower bound can further imply the lower bound of $\varOmega \left( \left( \frac{R}{\epsilon }\right) ^{\frac{d}{2\nu }}\left( \log \frac{R}{\epsilon }\right) ^{-1}\right)$, which nearly matches the upper bound, up to a replacement of *d*/2 by *d* and a logarithmic term $\log \frac{R}{\epsilon }$. However, when $\frac{\nu }{d}$ is small, there is still a significant gap between the lower bound implied by our general lower bound and the optimal lower bound.

##### Remark 5.4

There are two possible reasons why the bound is not tight. One potential reason is that we apply a conservative lower estimate for the metric entropy corresponding to the Matérn kernel. The other is that our metric entropy approach is limited in the regime of small smoothness parameter $\nu$. Filling this gap is left as future work.

## Experiments

In this section, we will first give a demonstration of adversarial functions, on which two common algorithms, the lower confidence bound (LCB)  [31] and the expected improvement (EI)  [13], perform poorly and achieve the optimization lower bound. Both algorithms model the unknown black-box function as sampled from a Gaussian process. The idea of LCB algorithm is minimizing the lower confidence bound, which is defined to be posterior mean minus a coefficient times the posterior standard deviation, to get the next sample point in each step. The EI algorithm maximizes the expected improvement with respect to the best observed value so far to get the next sample point. Then we run the two algorithms on a set of randomly sampled functions and compare the average performance and the adversarial performance in terms of simple regret. The algorithms are implemented based on GPy [11] and CasADi [1]. All the auxiliary optimization problems in the algorithms are solved using the solver IPOPT [35] with multiple different starting points. Our experiments take about 15 h on a device with AMD Ryzen Threadripper 3990X 64-Core Processor and 251 GB RAM.

### Demonstration of Adversarial Functions

In our proof of Theorem 5.1, we use a particular set of adversarial functions, which reveal value 0 to the algorithm and have low values somewhere else. In this section, we demonstrate such adversarial functions for two popular algorithms, expected improvement and lower confidence bound.

**Fig. 2:**
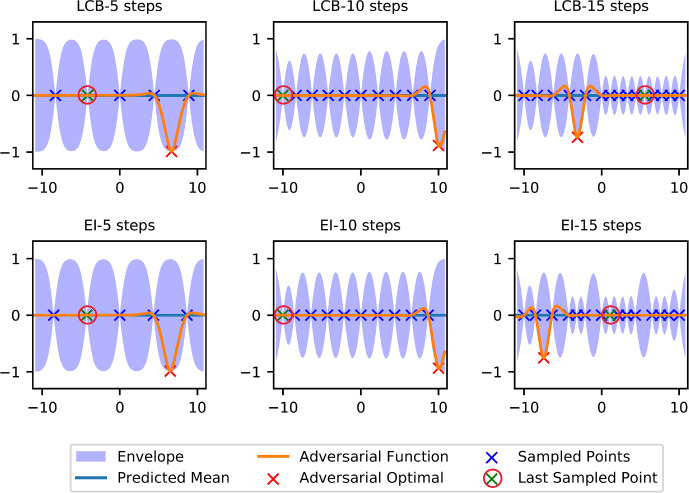
Demonstrations of adversarial functions in dimension one

We use the Matérn kernel in one dimension with $\nu =\frac{5}{2}, \rho =1, \sigma ^2=1$. We set the compact set to $\mathcal {X}=[-10, 10]$ and assume that the RKHS norm upper bound is $R=1$. We apply both lower confidence bound algorithm with the constant weight 1 for the posterior standard deviation and the expected improvement algorithm. We manually assign $x_1=0$ as the first sampled point and derive the adversarial function by solving Prob. (11).

11 $$\begin{aligned} \min _{x\in \mathcal {X}}\min _{s \in \mathcal {H}} ~s(x) \quad \text{ s.t. } \left\{ \begin{aligned}&s\left( x_{n}^0\right) =0,\;\; \forall \,n=1, \ldots , t, \\&\left\Vert s\right\Vert _\mathcal {H}\le R \end{aligned} \right. \end{aligned}$$

Thanks to the optimal recovery property [38, Thm 13.2], the optimal value for the inner problem of (11) can be analytically derived as

$$\begin{aligned} -R\sqrt{k(x,x)-k(x, X)^TK^{-1}k(X,x)}. \end{aligned}$$

Figure 2 demonstrates the adversarial functions inside the corresponding RKHS with a bounded norm of 1, which have value 0 at all the sampled points but have low global optimal value somewhere else. We notice that the envelope formed by the functions inside the ball with consistent evaluation data shrinks as more and more data becomes available. Intuitively, any algorithm needs to sample sufficiently densely globally in the adversarial case in order to find a close-to-optimal solution.

**Fig. 3:**
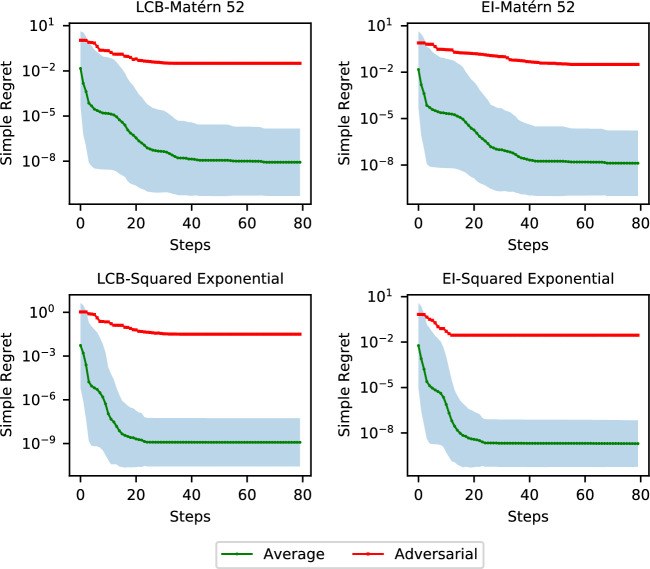
Comparison of average performance (±standard deviation shown as shaded area, over 100 instances) and adversarial performance. Adversarial simple regret is defined as the opposite of the optimal value of Prob. (11), namely the simple regret of the adversarial function at different optimization steps. Since the simple regret is defined as the best sampled function value minus the global optimal value (see definition 3.1), this plot can also be seen as the convergence rate plot if the algorithm reports the best sampled point

### Average vs. Adversarial Performance

The proofs of Theorems 5.2 and 5.3 indicate that a non-adaptive sampling algorithm can achieve a close-to-optimal worst-case convergence rate. However, in practice, adaptive algorithms (e.g., lower confidence bound and expected improvement) are usually adopted and perform better. There could potentially be a gap between average-case convergence and worst-case convergence. To perform such a comparison, we randomly sample a set of functions from the RKHS to run the algorithms over. Specifically, we first uniformly sample a finite set of knots $X\subset \mathcal {X}$ and then sample the function values $f_X$ on the knots from the marginal distribution of the Gaussian process, which is a finite-dimensional Gaussian distribution. We then construct the minimal norm interpolant of the knots as the sampled function. To be consistent with the bounded norm assumption, we reject the functions with a norm value larger than *R*.

We use simple regret, which is defined to be $\min _{\tau \in [t]}f(x_\tau )-\min _{x\in \mathcal {X}}f(x)$, to measure the performance of different algorithms. We set $\mathcal {X}=[0, 1]^3\subset \mathbb {R}^3$ and set the length scales and variances of both the Matérn kernel function (with $\nu =\frac{5}{2}$) and the squared exponential kernel. Figure 3 shows the comparison of average simple regret and adversarial simple regret. We observe that the average performance is much better than the performance on adversarial functions in terms of simple regret. Intuitively, adversarial functions are only a subset of *needle-in-haystack* functions, with most regions flat and somewhere very small, when *t* becomes large. For those adversarial functions such as shown in Fig. 2, it can be difficult for the efficient global optimization algorithms to “see” the trend of the function. For common functions inside the function space ball, however, the algorithms are still able to detect the trend of the function value and find a near-to-optimal solution quickly.

## Conclusions

In this paper, we provide a general lower bound on the worst-case suboptimality or simple regret for noiseless efficient global optimization in a non-Bayesian setting in terms of the metric entropy of the corresponding reproducing kernel Hilbert space (RKHS). We apply the general lower bounds to commonly used specific kernel functions, including the squared exponential kernel and the Matérn kernel. We further derive upper bounds and compare them to the lower bounds and find that they nearly match, except for the case for the Matérn kernel when $\frac{\nu }{d}$ is small. Two interesting future research directions are deriving an upper bound on the worst-case convergence rate in terms of metric entropy and characterizing the average-case convergence rate. We also conjecture that introducing randomness into the existing algorithms can improve the worst-case performance. An expected analysis challenge is that our current approach is sensitive to randomness. We also leave the extension of our analysis to the noisy case as future work.

## A Proof of Lemma 5.1

Consider the optimization problem below,

12 $$\begin{aligned} \min _{s \in \mathcal {H}} \;\;\Vert s\Vert _{\mathcal {H}}^{2} \quad \text{ s.t. } \;\; s\left( x_{n}\right) =f(x_{n}), \;\; \forall \, n=1, \ldots , t. \end{aligned}$$

Based on the representer theorem [36, Theorem 1.3.1], the optimal solution of (12) has the form $\sum _{i=1}^t\alpha _ik(x_i, \cdot )$. By using the constraint $s(x_n)=f(x_n)$, we can derive $K\alpha =f_X$, where $f_X=[f(x_1), f(x_2), \cdots , f(x_n)]^T$ and $K=(k(x_i,x_j))_{i\in [n], j\in [n]}$. With this restriction, we transform the problem in (12) to the problem in (13).

13 $$\begin{aligned} \underset{\alpha \in \mathbb {R}^t}{ \min } \;\;~\alpha ^TK\alpha \quad \text{ s.t. } \;\; K\alpha =f_X`. \end{aligned}$$

We take $\alpha ^*$ as the solution to the problem in (13), whose feasibility is guaranteed by representer theorem [36] and the non-emptiness of the feasible set (*f* is feasible for (12)). Therefore, $m_t(x)=(\alpha ^*)^TK_{Xx}$ is the optimal solution to (12). Since *f* is a feasible solution for the problem (12), $\left\Vert m_t\right\Vert _\mathcal {H}\le \left\Vert f\right\Vert _\mathcal {H}\le R$. In addition, $(f-m_t)(x_i)=f(x_i)-m_t(x_i)=0, \forall i\in [t]$. And $\left\Vert f-m_t\right\Vert _\mathcal {H}^2=\left\Vert f\right\Vert _\mathcal {H}^2+\left\Vert m_t\right\Vert _\mathcal {H}^2-2\langle f, m_t\rangle =\left\Vert f\right\Vert _\mathcal {H}^2-\left\Vert m_t\right\Vert _\mathcal {H}^2\le R^2$. So $m_t\in S_t^\Vert$ and $f-m_t\in S_t^\perp$.

## B Proof of Lemma 5.2

Let $(p_1,p_2,\ldots ,p_m)$ be an $\epsilon _t^\Vert$-covering of $S_t^\Vert (\mathcal {X})$ and $(q_1,q_2,\ldots ,q_n)$ an $\epsilon _t^\perp$-covering of $S_t^\perp (\mathcal {X})$. Then $\forall f\in S$, by Lemma 5.1, $f=m_t+(f-m_t)$, where $m_t\in S_t^\Vert$ and $f-m_t\in S_t^\perp$. By the definition of covering, $\exists p_i$, such that $\left\Vert m_t|_{\mathcal {X}}-p_i\right\Vert _\infty \le \epsilon _t^\Vert$ and $\exists q_j$, such that $\left\Vert (f-m_t)|_{\mathcal {X}}-q_j\right\Vert _\infty \le \epsilon _t^\perp$. So

$$\begin{aligned} \left\Vert f|_\mathcal {X}-(p_i+q_j)\right\Vert _\infty \le \left\Vert m_t|_\mathcal {X}-p_i\right\Vert _\infty +\left\Vert (f-m_t)|_\mathcal {X}-q_j\right\Vert _\infty \le \epsilon _t^\Vert +\epsilon _t^\perp =\epsilon _t. \end{aligned}$$

So the set $\{p_i+q_j|i\in [m],j\in [n]\}$ is an $\epsilon _t$-covering of $S(\mathcal {X})$ and we have the cardinality

$$\begin{aligned} |\{p_i+q_j|i\in [m],j\in [n]\}|= & {} \mathcal {N}(S_t^\perp (\mathcal {X}), \epsilon _t^\perp , \left\Vert \cdot \right\Vert _\infty )\mathcal {N}(S_t^\Vert (\mathcal {X}), \epsilon _t^\Vert , \left\Vert \cdot \right\Vert _\infty )\\\ge & {} \mathcal {N}(S(\mathcal {X}), \epsilon _t,\left\Vert \cdot \right\Vert _\infty ). \end{aligned}$$

So $$\mathcal {M}(S_t^\perp (\mathcal {X}), \epsilon _t^\perp , \left\Vert \cdot \right\Vert _\infty )\ge \mathcal {N}(S_t^\perp (\mathcal {X}), \epsilon _t^\perp , \left\Vert \cdot \right\Vert _\infty ) \ge \frac{\mathcal {N}(S(\mathcal {X}), \epsilon _t,\left\Vert \cdot \right\Vert _\infty )}{\mathcal {N}(S_t^\Vert (\mathcal {X}), \epsilon _t^\Vert ,\left\Vert \cdot \right\Vert _\infty )}$$.

## C Proof of Lemma 5.3

We first introduce the set, $\mathcal {E}_t=\{\alpha \in \mathbb {R}^t|\alpha ^T K_t\alpha \le R^2\}$, where $K_t=(k(x_i,x_j))_{i,j\in [t]}$. Without loss of generality, we assume that $K_t$ has full rank in the following analysis. Notice that if this condition does not hold, we only need to restrict to the subspace spanned by the eigenvectors of $K_t$ with strictly positive eigenvalues and consider the intersection of $\mathcal {E}_t$ with the subspace. Since the restriction only reduces the essential dimension, the upper bound still holds. We introduce the norm $\left\Vert \alpha \right\Vert _{K_t}=\sqrt{\alpha ^TK_t\alpha }$. We then have, $\forall f(x)=\alpha ^Tk(X,x)\in S_t^\Vert (\mathcal {X}), g(x)=\beta ^Tk(X,x)\in S_t^\Vert (\mathcal {X})$, we have

14 $$\begin{aligned}&\left\Vert f-g\right\Vert _\infty =~\sup _{x\in \mathcal {X}}|(\alpha -\beta )^Tk(X,x)| =~\sup _{x\in \mathcal {X}}|\left\langle \sum _{i\in [t]}(\alpha _i-\beta _i)k(x_i,\cdot ), k(x,\cdot )\right\rangle |\nonumber \\&\quad \le \! \sup _{x\in \mathcal {X}}\left\Vert \sum _{i\in [t]}(\alpha _i\!-\!\beta _i)k(x_i,\cdot )\right\Vert _\mathcal {H}\!\left\Vert k(x,\cdot )\right\Vert _\mathcal {H}\!\le \! \sup _{x\in \mathcal {X}}\left\Vert \alpha \!-\!\beta \right\Vert _{K_t}\sqrt{k(x,x)}\!\le \!\left\Vert \alpha \!-\!\beta \right\Vert _{K_t}\!. \end{aligned}$$

Therefore, we have $\mathcal {N}(S^\Vert _t(\mathcal {X}), \epsilon ,\left\Vert \cdot \right\Vert _\infty )\le \mathcal {N}(\mathcal {E}_t, \epsilon ,\left\Vert \cdot \right\Vert _{K_t})$. We further have,

15a $$\begin{aligned}&\mathcal {N}(\mathcal {E}_t, \epsilon ,\left\Vert \cdot \right\Vert _{K_t}) \le ~\mathcal {M}(\mathcal {E}_t, \epsilon ,\left\Vert \cdot \right\Vert _{K_t})\le ~\frac{\textrm{Vol}\left( B_{\left\Vert \cdot \right\Vert _{K_t}}\left( 0, R+\frac{\epsilon }{2}\right) \right) }{\textrm{Vol}\left( B_{\left\Vert \cdot \right\Vert _{K_t}}\left( 0, \frac{\epsilon }{2}\right) \right) } \end{aligned}$$

15b $$\begin{aligned}&\quad =~\left( \frac{2R}{\epsilon }+1\right) ^t\le ~\left( \frac{R^2}{2\epsilon ^2}+\frac{R^2}{2\epsilon ^2}\right) ^t =~\left( \frac{R}{\epsilon }\right) ^{2t}. \end{aligned}$$

The second inequality in (15) follows by that if $\alpha _1, \alpha _2,\ldots ,\alpha _M$ is an $\epsilon$-packing of the set $\mathcal {E}_t$, then $\cup _{i\in [M]}B_{\left\Vert \cdot \right\Vert _{K_t}}\left( \alpha _i, \frac{\epsilon }{2}\right) \subset B_{\left\Vert \cdot \right\Vert _{K_t}}\left( 0, R+\frac{\epsilon }{2}\right)$ and $B_{\left\Vert \cdot \right\Vert _{K_t}}\left( \alpha _i, \frac{\epsilon }{2}\right) \cap B_{\left\Vert \cdot \right\Vert _{K_t}}\left( \alpha _j, \frac{\epsilon }{2}\right) =\emptyset ,\forall i\ne j$ by the definition of packing. The third inequality in (15) follows by the assumption of $0<\epsilon <\frac{R}{4}$. So $\log \mathcal {N}(\mathcal {E}_t, \epsilon ,\left\Vert \cdot \right\Vert _{K_t})\le 2t\log \left( \frac{R}{\epsilon }\right)$. Therefore, $\log \mathcal {N}(S^\Vert _t(\mathcal {X}), \epsilon ,\left\Vert \cdot \right\Vert _\infty )\le 2t\log \left( \frac{R}{\epsilon }\right)$.

## D Proof of Theorem 5.2

By [42, Example 1], the covering number satisfies,

16 $$\begin{aligned} \log \mathcal {N}(S(\mathcal {X}), 4\epsilon , \left\Vert \cdot \right\Vert _\infty )=\varOmega \left( \log \left( \frac{R}{\epsilon }\right) ^{\frac{d}{2}}\right) . \end{aligned}$$

Therefore, Theorem 5.1 implies that,

17 $$\begin{aligned} T = \varOmega \left( \left( \log \frac{R}{\epsilon }\right) ^{\frac{d}{2}-1}\right) . \end{aligned}$$

We now focus on proving the upper bound part. To facilitate the following proof, we define,

18

19

where $m_t(x)=f_X^TK^{-1}K_{Xx}$ and $\sigma _t(x)=\sqrt{k(x,x)-K_{xX}K^{-1}K_{Xx}}$. Note that with squared exponential kernel and the sampled points set *X* to be used in this proof, the invertibility of the matrix *K* is guaranteed. As implied by [19, Prop. 1],

20

We consider the algorithm that evaluates the grid points

$$\begin{aligned} X=\left. \left\{ \left( \frac{k_1}{N}, \frac{k_2}{N},\ldots , \frac{k_d}{N}\right) \right| k_i\in \{0,1,\ldots , N-1\}\right\} \end{aligned}$$

without adaptation, and evaluate the point ${\tilde{x}}_t$ before termination after $t=N^d$ function evaluations on the grid points, where ${\tilde{x}}_t$ is given as,

21

Let $x^*$ denote the ground-truth optimal solution. We can bound the suboptimality,

22a

22b

22c

22d

22e

where the inequalities in (22a) and (22b) follow by (20) and the inequality (22c) follows by the definition of ${\tilde{x}}_t$ in (21). We now try to upper bound $\sigma _t({\tilde{x}}_t)$. We first introduce a set of Lagrangian interpolation functions,

$$\begin{aligned} w_{\alpha ,N}(x)=\prod _{i\in [N]}\prod _{j\in [N],j\ne \alpha _i}\frac{x_i-j/N}{\alpha _i/N-j/N}, x\in [0,1]^d, \alpha \in [N]^d. \end{aligned}$$

Let $w_N(x)=\left( w_{\alpha ,N}(x)\right) _{\alpha \in [N]^d}$ and $\beta _N(x)=K^{-1}K_{Xx}\in \mathbb {R}^{N^d}$, we have

23a $$\begin{aligned} (\sigma _{t}(x))^2=&~k(x,x)-K_{xX}K^{-1}K_{Xx} \end{aligned}$$

23b $$\begin{aligned} =&~k(x,x)-2K_{xX}\beta _N(x)+\beta _N(x)^TK\beta _N(x) \end{aligned}$$

23c $$\begin{aligned} =&~\min _{y\in \mathbb {R}^{N^d}}(k(x,x)-2K_{xX}y+y^TKy) \end{aligned}$$

23d $$\begin{aligned} \le&~k(x,x)-2K_{xX}w_N(x)+w_N(x)^TKw_N(x). \end{aligned}$$

Let $k_0(x)$ denote the function *k*(0, *x*) and ${\hat{k}}_0$ its corresponding Fourier transformation. By inverse Fourier transformation, we have,

24a $$\begin{aligned}&k(x,x)-2K_{xX}w_N(x)+w_N(x)^TKw_N(x) \end{aligned}$$

24b $$\begin{aligned}&\quad =~(2 \pi )^{-d} \int _{\mathbb {R}^{d}} {\hat{k}}_0(\xi )\left( 1-2\sum _{\alpha \in {[N]}^d} w_{\alpha , N}(x) e^{i \xi \cdot \left( x-\frac{\alpha }{N}\right) }\right. \end{aligned}$$

24c $$\begin{aligned}&\qquad +\left. \sum _{\alpha \in {[N]}^d, \beta \in {[N]}^d} w_{\alpha , N}(x) e^{i \xi \cdot \left( \frac{\beta }{N}-\frac{\alpha }{N}\right) }w_{\beta , N}(x)\right) \mathop {}\!\textrm{d}\xi \end{aligned}$$

24d $$\begin{aligned}&\quad =~(2 \pi )^{-d} \int _{\mathbb {R}^{d}} {\hat{k}}_0(\xi )\left| 1-\sum _{\alpha \in {[N]}^d} w_{\alpha , N}(x) e^{i \xi \cdot \left( x-\frac{\alpha }{N}\right) }\right| ^{2} \mathop {}\!\textrm{d}\xi \end{aligned}$$

24e $$\begin{aligned}&\quad =~(2 \pi )^{-d} \int _{\mathbb {R}^{d}} {\hat{k}}_0(\xi )\left| e^{-i \frac{\xi }{N} \cdot N x}-\sum _{\alpha \in [N]^d} w_{\alpha , N}(x) e^{-i \frac{\xi }{N} \cdot \alpha }\right| ^{2} \mathop {}\!\textrm{d}\xi \end{aligned}$$

24f $$\begin{aligned}&\quad = (2 \pi )^{-d} \int _{\mathbb {R}^{d}} {\hat{k}}_0(\xi )\left| e^{-i \frac{\xi }{N} \cdot N x}-\sum _{\alpha \in [N]^d} w_{\alpha , N}(x) e^{-i \frac{\xi }{N} \cdot \alpha }\right| ^{2} \mathop {}\!\textrm{d}\xi \end{aligned}$$

24g $$\begin{aligned}&\quad = (2 \pi )^{-d} \int _{\xi \in [-\frac{N}{2}, \frac{N}{2}]^d} {\hat{k}}_0(\xi )\left| e^{-i \frac{\xi }{N} \cdot N x}-\sum _{\alpha \in [N]^d} w_{\alpha , N}(x) e^{-i \frac{\xi }{N} \cdot \alpha }\right| ^{2} \mathop {}\!\textrm{d}\xi \end{aligned}$$

24h $$\begin{aligned}&\qquad +(2 \pi )^{-d} \int _{\xi \not \in [-\frac{N}{2}, \frac{N}{2}]^d} {\hat{k}}_0(\xi )\left| e^{-i \frac{\xi }{N} \cdot N x}-\sum _{\alpha \in [N]^d} w_{\alpha , N}(x) e^{-i \frac{\xi }{N} \cdot \alpha }\right| ^{2} \mathop {}\!\textrm{d}\xi . \end{aligned}$$

To proceed, we need to use the Lemma D.1 [41].

### Lemma D.1

(Lemma 4.1, [41]) Let $x \in [0,1]^{d}$ and $N \in \mathbb {N}$. Then $\sum _{\alpha \in [N]^d}\left| w_{\alpha , N}(x)\right| \le \left( N 2^{N}\right) ^{d}$ and for $\theta \in \left[ -\frac{1}{2}, \frac{1}{2}\right] ^{d}$, there holds

$$\begin{aligned} \left| e^{-i \theta \cdot N x}-\sum _{\alpha \in {[N]^d}} w_{\alpha , N}(x) e^{-i \theta \cdot \alpha }\right| \le d\left( 1+\frac{1}{2^{N}}\right) ^{d-1}\left( \max _{1 \le j\le d}\left| \theta _{j}\right| \right) ^{N}. \end{aligned}$$

We apply the bounds in Lemma D.1 to Eq. (24) and have,

25a $$\begin{aligned}&k(x,x)-2K_{xX}w_N(x)+w_N(x)^TKw_N(x) \end{aligned}$$

25b $$\begin{aligned}&\quad =~(2 \pi )^{-d} \int _{\xi \in [-\frac{N}{2}, \frac{N}{2}]^d} {\hat{k}}_0(\xi )\left| e^{-i \frac{\xi }{N} \cdot N x}-\sum _{\alpha \in [N]^d} w_{\alpha , N}(x) e^{-i \frac{\xi }{N} \cdot \alpha }\right| ^{2} \mathop {}\!\textrm{d}\xi \end{aligned}$$

25c $$\begin{aligned}&\qquad ~+(2 \pi )^{-d} \int _{\xi \not \in [-\frac{N}{2}, \frac{N}{2}]^d} {\hat{k}}_0(\xi )\left| e^{-i \frac{\xi }{N} \cdot N x}-\sum _{\alpha \in [N]^d} w_{\alpha , N}(x) e^{-i \frac{\xi }{N} \cdot \alpha }\right| ^{2} \mathop {}\!\textrm{d}\xi \end{aligned}$$

25d $$\begin{aligned}&\quad \le ~d\left( 1+\frac{1}{2^{N}}\right) ^{d-1} \max _{1 \le j \le d}\left\{ (2 \pi )^{-d} \int _{\xi \in \left[ -\frac{N}{2}, \frac{N}{2}\right] ^{d}} {\hat{k}}_0(\xi )\left( \frac{\left| \xi _{j}\right| }{N}\right) ^{N} \mathop {}\!\textrm{d}\xi \right\} \end{aligned}$$

25e $$\begin{aligned}&\qquad +\frac{\left( 1+\left( N 2^{N}\right) ^{d}\right) ^{2}}{(2 \pi )^{d}} \int _{\xi \notin \left[ -\frac{N}{2}, \frac{N}{2}\right] ^{d}} {\hat{k}}_0(\xi ) \mathop {}\!\textrm{d}\xi . \end{aligned}$$

We know that ${\hat{k}}_0(\xi )=(\sigma \sqrt{\pi })^{d} e^{-\frac{\sigma ^{2}|\xi |^{2}}{4}}$. Similar to the analysis in the proof of Example 4 of [41], we first try to bound the first term in the upper bound derived in Eq. (25).

26a $$\begin{aligned}&(2 \pi )^{-d} \int _{\xi \in \left[ -\frac{N}{2}, \frac{N}{2}\right] ^{d}}(\sigma \sqrt{\pi })^{d} e^{-\frac{\sigma ^{2}|\xi |^{2}}{4}}\left( \frac{\left| \xi _{j}\right| }{N}\right) ^{N} \mathop {}\!\textrm{d}\xi \end{aligned}$$

26b $$\begin{aligned}&\quad =\frac{\sigma \sqrt{\pi }}{2\pi } \int _{-N/2}^{N/2} e^{-\frac{\sigma ^{2}\xi _j^{2}}{4}}\left( \frac{\left| \xi _{j}\right| }{N}\right) ^{N} \left( \prod _{k\ne j}\int _{-N/2}^{N/2}\frac{\sigma \sqrt{\pi }}{2\pi } e^{-\frac{\sigma ^{2}\xi _k^{2}}{4}}\mathop {}\!\textrm{d}\xi _k\right) \mathop {}\!\textrm{d}\xi _j \end{aligned}$$

26c $$\begin{aligned}&\quad \le ~\frac{\sigma \sqrt{\pi }}{2 \pi } \int _{-N / 2}^{N / 2} e^{-\frac{\sigma ^{2}\left| \xi _{j}\right| ^{2}}{4}}\left( \frac{\left| \xi _{j}\right| }{N}\right) ^{N} \mathop {}\!\textrm{d}\xi _{j} \le ~\frac{2}{\sqrt{\pi }}\left( \frac{2}{\sigma N}\right) ^{N} \varGamma \left( \frac{N+1}{2}\right) , \end{aligned}$$

where $\varGamma (\cdot )$ is the Gamma function. The first inequality in (26) follows by that

$$\begin{aligned} \int _{-N/2}^{N/2}\frac{\sigma \sqrt{\pi }}{2\pi } e^{-\frac{\sigma ^{2}\xi _k^{2}}{4}}\mathop {}\!\textrm{d}\xi _k\le \int _{-\infty }^{+\infty }\frac{\sigma \sqrt{\pi }}{2\pi } e^{-\frac{\sigma ^{2}\xi _k^{2}}{4}}\mathop {}\!\textrm{d}\xi _k=1 \end{aligned}$$

and the second inequality in (26) follows by that

$$\begin{aligned} \int _{-N / 2}^{N / 2} e^{-\frac{\sigma ^{2}\left| \xi _{j}\right| ^{2}}{4}}\left( \frac{\left| \xi _{j}\right| }{N}\right) ^{N} \mathop {}\!\textrm{d}\xi _{j}&=2\int _{0}^{N / 2} e^{-\frac{\sigma ^{2}\left| \xi _{j}\right| ^{2}}{4}}\left( \frac{\left| \xi _{j}\right| }{N}\right) ^{N} \mathop {}\!\textrm{d}\xi _{j}\\&\le 2\int _{0}^{+\infty } e^{-\frac{\sigma ^{2}\left| \xi _{j}\right| ^{2}}{4}}\left( \frac{\left| \xi _{j}\right| }{N}\right) ^{N} \mathop {}\!\textrm{d}\xi _{j} \end{aligned}$$

and the definition of Gamma function. Applying Stirling’s formula yields

27a $$\begin{aligned}&(2 \pi )^{-d} \int _{\xi \in \left[ -\frac{N}{2}, \frac{N}{2}\right] ^{d}}(\sigma \sqrt{\pi })^{d} e^{-\frac{\sigma ^{2}|\xi |^{2}}{4}}\left( \frac{\left| \xi _{j}\right| }{N}\right) ^{N} \mathop {}\!\textrm{d}\xi \end{aligned}$$

27b $$\begin{aligned}&\quad \le ~\frac{2}{\sqrt{\pi }}\left( \frac{2}{\sigma N}\right) ^{N} \varGamma \left( \frac{N+1}{2}\right) \le ~2\left( \frac{2}{\sigma N}\right) ^{N}\left( \frac{N+1}{2 e}\right) ^{\frac{N+1}{2}} \frac{1}{\sqrt{N+1}} e^{\frac{1}{6(N+1)}} \end{aligned}$$

27c $$\begin{aligned}&\quad =~\sqrt{\frac{2}{e}}e^{\frac{1}{6(N+1)}}\left( \frac{2\sqrt{\frac{N+1}{2}}}{\sigma \sqrt{e}N}\right) ^N \le ~\sqrt{2e}\left( \frac{{2}}{\sigma \sqrt{e N}}\right) ^{N}, \end{aligned}$$

where the second inequality in (27) follows by the Stirling’s formula that

$$\begin{aligned} \varGamma (u)\le \sqrt{2\pi }u^{u-\frac{1}{2}}e^{-u}e^{\frac{1}{12u}},\quad u>0 \end{aligned}$$

and the last inequality follows by $e^{\frac{1}{6(N+1)}}\le e$ and $\frac{N+1}{2}\le N$. We are now to bound the second term in Eq. (24) as follows,

28 $$\begin{aligned}&(2 \pi )^{-d} \int _{\xi \notin \left[ -\frac{N}{2}, \frac{N}{2}\right] ^{d}}(\sigma \sqrt{\pi })^{d} e^{-\frac{\sigma ^{2}|\xi |^{2}}{4}} \mathop {}\!\textrm{d}\xi =~\left( \frac{\sigma \sqrt{\pi }}{2 \pi } \int _{\xi _{j} \notin [-\frac{N}{2}, \frac{N}{2}]} e^{-\frac{\sigma ^{2}\left| \xi _{j}\right| ^{2}}{4}} \mathop {}\!\textrm{d}\xi _{j} \right) ^d\nonumber \\&\quad = \left( \frac{\sigma \sqrt{\pi }}{\pi } \int _{N / 2}^{+\infty } e^{-\frac{\sigma ^{2}}{4}\left( t^{2}-t / 2\right) } e^{-\frac{\sigma ^{2}}{4} \cdot \frac{t}{2}} \mathop {}\!\textrm{d}t\right) ^d\nonumber \\&\quad \le ~\left( \frac{\sigma \sqrt{\pi }}{\pi } \int _{N / 2}^{+\infty } e^{-\frac{\sigma ^{2}}{4}\left( \left( \frac{N}{2}\right) ^{2}-\left( \frac{N}{4}\right) \right) } e^{-\frac{\sigma ^{2}}{4} \cdot \frac{t}{2}} \mathop {}\!\textrm{d}t\right) ^d\nonumber \\&\quad = \left( \frac{\sigma }{\sqrt{\pi }} e^{-\frac{\sigma ^{2} N(N-1)}{16}} \frac{8}{\sigma ^{2}} e^{-\frac{\sigma ^{2} N}{16}}\right) ^d=~\left( \frac{8}{\sigma \sqrt{\pi }}\right) ^d e^{-\frac{\sigma ^{2}}{16}dN^{2}}. \end{aligned}$$

Combining (24), (27) and (28) yields

29a $$\begin{aligned}&k(x,x)-2K_{xX}w_N(x)+w_N(x)^TKw_N(x) \end{aligned}$$

29b $$\begin{aligned}&\quad \le ~\sqrt{2e}d\left( 1+\frac{1}{2^{N}}\right) ^{d-1} \left( \frac{{2}}{\sigma \sqrt{e N}}\right) ^{N}+\left( 1+\left( N 2^{N}\right) ^{d}\right) ^{2}\left( \frac{8}{\sigma \sqrt{\pi }}\right) ^d e^{-\frac{\sigma ^{2}}{16}dN^{2}} \end{aligned}$$

29c $$\begin{aligned}&\quad \le ~\sqrt{2e}d2^{d-1}\left( \frac{{2}}{\sigma \sqrt{e N}}\right) ^{N}+4\left( N 2^{N}\right) ^{2d}\left( \frac{8}{\sigma \sqrt{\pi }}\right) ^d e^{-\frac{\sigma ^{2}}{16}dN^{2}} \end{aligned}$$

29d $$\begin{aligned}&\quad =~\sqrt{2e}d2^{d-1}\left( \frac{{2}}{\sigma \sqrt{e N}}\right) ^{N}+4\left( \frac{8}{\sigma \sqrt{\pi }}\right) ^d e^{-\frac{\sigma ^{2}}{16}dN^{2}+2d(N\log 2+\log N)} \end{aligned}$$

29e $$\begin{aligned}&\quad \le ~\sqrt{2e}d2^{d-1}\left( \frac{{2}}{\sigma \sqrt{e N}}\right) ^{N}+4\left( \frac{8}{\sigma \sqrt{\pi }}\right) ^d e^{-\frac{\sigma ^{2}}{16}dN^{2}+2d(\log 2+1) N}, \end{aligned}$$

where the first inequality follows by combining (24), (27) and (28), the second inequality follows by that $1+\frac{1}{2^N}\le 2$ and $1+(N2^N)^d\le 2(N2^N)^d$, and the last inequality follows by that $\log N\le N$. Let $N\ge \max \{\frac{32(\log 2+2)}{\sigma ^2}, \frac{4e^{2d-1}}{\sigma ^2}\}$, we have

30a $$\begin{aligned}&k(x,x)-2K_{xX}w_N(x)+w_N(x)^TKw_N(x) \end{aligned}$$

30b $$\begin{aligned}&\quad \le ~\sqrt{2e}d2^{d-1}\left( \frac{{2}}{\sigma \sqrt{e N}}\right) ^{N}+4\left( \frac{8}{\sigma \sqrt{\pi }}\right) ^d e^{-\frac{\sigma ^{2}}{16}dN^{2}+2d(\log 2+1) N} \end{aligned}$$

30c $$\begin{aligned}&\quad \le ~\sqrt{2e}d2^{d-1}\left( e^{-d}\right) ^{N}+4\left( \frac{8}{\sigma \sqrt{\pi }}\right) ^d e^{-dN} \end{aligned}$$

30d $$\begin{aligned}&\quad =~\left( \sqrt{2e}d2^{d-1}+4\left( \frac{8}{\sigma \sqrt{\pi }}\right) ^d\right) e^{-dN}. \end{aligned}$$

Combining (23), (30) and that $N^d=t$, we have

31 $$\begin{aligned} \sigma _t(x)\le \left( \sqrt{2e}d2^{d-1}+4\left( \frac{8}{\sigma \sqrt{\pi }}\right) ^d\right) ^{\frac{1}{2}}e^{-\frac{1}{2}dt^{1/d}}, \forall x\in [0,1]^d. \end{aligned}$$

Combining that $f({\tilde{x}}_t)-\min _{x\in \mathcal {X}}f(x)\le 2R\sigma _t({\tilde{x}}_t)$ in (22) and (31), we have

32 $$\begin{aligned} f({\tilde{x}}_t)-\min _{x\in \mathcal {X}}f(x)\le 2R\left( \sqrt{2e}d2^{d-1}+4\left( \frac{8}{\sigma \sqrt{\pi }}\right) ^d\right) ^{\frac{1}{2}}e^{-\frac{1}{2}dt^{1/d}}. \end{aligned}$$

Setting the right hand side to be smaller than $\epsilon$, we observe that the number of steps *t* only needs to be $\mathcal {O}\left( \left( \log \frac{R}{\epsilon }\right) ^d\right)$. This completes the proof.

## E Proof of Theorem 5.3

By Lemma 3 in [4], the RKHS on $[0, 1]^d$ is equivalent to Sobolev Hilbert space $H^{\nu +\frac{d}{2}}((0,1)^d)$. Implied by [8, Theorem 1, Sect. 3.3.3], the covering number of the function space ball in $H^{\nu +\frac{d}{2}}((0, 1)^d)$ is lower bounded by $\varOmega \left( \left( \frac{R}{\epsilon }\right) ^{\frac{d}{\nu +d/2}}\right)$. Therefore,

33 $$\begin{aligned} \log \mathcal {N}(S(\mathcal {X}), 4\epsilon , \left\Vert \cdot \right\Vert _\infty )=\varOmega \left( \left( \frac{R}{\epsilon }\right) ^{\frac{d}{\nu +d/2}}\right) . \end{aligned}$$

We then apply Theorem 5.1 such that we can get the lower bound

34 $$\begin{aligned} T=\varOmega \left( \left( \frac{R}{\epsilon }\right) ^{\frac{d}{\nu +d/2}}\left( \log \left( \frac{R}{\epsilon }\right) \right) ^{-1}\right) . \end{aligned}$$

The upper bound is implied by Theorem 1 in [4].

## Footnotes

1 The Bayesian setting is typically referred to as Bayesian optimization.

2 We will use $\epsilon$ to denote the desired accuracy throughout the paper.

3 In the Gaussian process bandit literature, the maximizition formulation is usually adopted, while in the global optimization literature, the minimization formulation is usually adopted. Here, we adopt the latter.
